# Nrf2-mediated epigenetic priming of osmoprotective genes enhances cellular adaptation to hyperosmotic stress

**DOI:** 10.1016/j.jbc.2026.113278

**Published:** 2026-06-23

**Authors:** Yi Lu, Na Li, Xudan Dou, Ziling Zhang, Yongxuan Su, Xueting Li, Jing Wang, Weiping Tian, Shao-Kai Sun, Lirong Zhang, Yupeng Chen, Zhiheng Liu

**Affiliations:** 1 The Province and Ministry Co-sponsored Collaborative Innovation Center for Medical Epigenetics, Key Laboratory of Immune Microenvironment and Disease (Ministry of Education), State Key Laboratory of Experimental Hematology, Department of Biochemistry and Molecular Biology, School of Basic Medical Sciences, Tianjin Medical University, Tianjin, China; 2 School of Medical Imaging, Tianjin Key Laboratory of Functional Imaging, Tianjin Medical University, Tianjin, China; 3 Research Center of Basic Medical Sciences, Tianjin Medical University, Tianjin, China; 4 Department of Urology, Tianjin Institute of Urology, The Second Hospital of Tianjin Medical University, Tianjin, China

**Keywords:** hyperosmotic stress, kidney, NFAT5, Nrf2, osmoprotective genes

## Abstract

Hyperosmotic stress triggers a complex adaptive response that enables cells to maintain homeostasis and survive osmotic perturbations. However, the molecular mechanisms that coordinate transcriptional and epigenetic programs in response to osmotic stress remain poorly defined. Here, through an unbiased chemical screen, we identify activation of nuclear factor erythroid 2 - related factor 2 (Nrf2) as a potent enhancer of cell survival under hyperosmotic stress. Mechanistically, Nrf2 does not function as a sustained transcriptional activator of osmoprotective genes during stress. Instead, Nrf2 establishes a primed chromatin state prior to osmotic challenge, characterized by increased enrichment of activation-associated histone modifications at osmoprotective loci. This epigenetic priming enables enhanced recruitment of NFAT5 upon hyperosmotic stimulation, thereby amplifying osmoprotective gene transcription. Disruption of Nrf2 abolishes chromatin activation, NFAT5 binding, and transcriptional induction of osmoprotective genes, whereas pharmacological Nrf2 activation restores these processes and improves cell survival. In a model of dehydration-induced hyperosmotic stress, renal cell death was markedly increased in *Nrf2*-deficient mice, while Nrf2 activation promoted the expression of osmoprotective genes and conferred tissue protection. Together, our findings identify Nrf2 as an epigenetic priming factor that licenses NFAT5 - dependent transcription under hyperosmotic stress, revealing a previously unrecognized chromatin-based mechanism that enhances cellular adaptation to osmotic challenges.

Hyperosmotic stress elicits a complex adaptive response that enables cells to preserve homeostasis and survive osmotic perturbations (1, 2, 3). This challenge is particularly relevant in pathological settings such as chronic dehydration or diabetes-associated hyperglycemia, where tissues are persistently exposed to elevated extracellular osmolality, leading to cellular injury, inflammation, and progressive organ dysfunction (4, 5, 6, 7). Despite its physiological and pathological importance, the molecular mechanisms that coordinate cellular adaptation to hyperosmotic stress remain incompletely understood.

The transcriptional response to hyperosmotic stress is coordinated by multiple regulatory factors, among which nuclear factor of activated T cells 5 (NFAT5, also known as TonEBP/OREBP) plays a central and well-established role (8). When cells are exposed to elevated extracellular osmolality, water efflux leads to cell shrinkage and increased intracellular ionic strength. These biophysical changes promote NFAT5 activation through enhanced protein stability, post-translational modification, and nuclear translocation. Activated NFAT5 binds to osmotic response elements within target gene promoters and induces the expression of key osmoprotective genes, thereby facilitating osmolyte accumulation to restore cell volume and maintain cellular function (9, 10, 11). However, NFAT5-mediated transcription represents only part of the broader osmoadaptive response. How NFAT5-dependent gene regulation is integrated with other transcriptional and epigenetic mechanisms, particularly under sustained or pathological hyperosmotic stress, remains poorly defined.

To identify regulatory pathways that enhance cellular survival under hyperosmotic conditions, we performed an unbiased high-throughput chemical screen for modulators of osmotic stress - induced cell death. This approach revealed a marked enrichment of compounds that activate nuclear factor erythroid 2 - related factor 2 (Nrf2). Nrf2 is classically recognized as a master regulator of antioxidant and cytoprotective gene expression (12); however, whether and how Nrf2 participates in the regulation of osmotic stress-responsive transcription has not been systematically explored.

Given the canonical role of Nrf2 as a transcriptional regulator, several non-mutually exclusive mechanisms could potentially explain how Nrf2 activation enhances cellular tolerance to hyperosmotic stress. Nrf2 might directly activate osmotic stress-responsive genes, or it could potentiate the activity of the central osmotic stress transcription factor NFAT5 by influencing its expression or activation. Alternatively, Nrf2 could modulate chromatin states that define transcriptional competence, thereby shaping the magnitude and robustness of stress-induced transcriptional responses.

In this study, we systematically evaluate these possibilities by integrating high-throughput chemical screening with transcriptomic profiling, chromatin state mapping, and *in vivo* analysis. Our data support a model in which Nrf2 enhances osmotic stress tolerance primarily by functioning as an epigenetic priming factor. By establishing a permissive chromatin landscape at osmoprotective gene loci prior to osmotic challenge, Nrf2 licenses efficient NFAT5 recruitment and amplifies NFAT5-dependent transcription upon stress exposure. Together, these findings reveal a previously unrecognized layer of gene regulation in osmotic stress adaptation and identify chromatin priming as a key determinant of stress-induced transcriptional output.

## Results

### Chemical screening identified Nrf2 activators as protective agents against hyperosmotic stress

**Figure 1:**
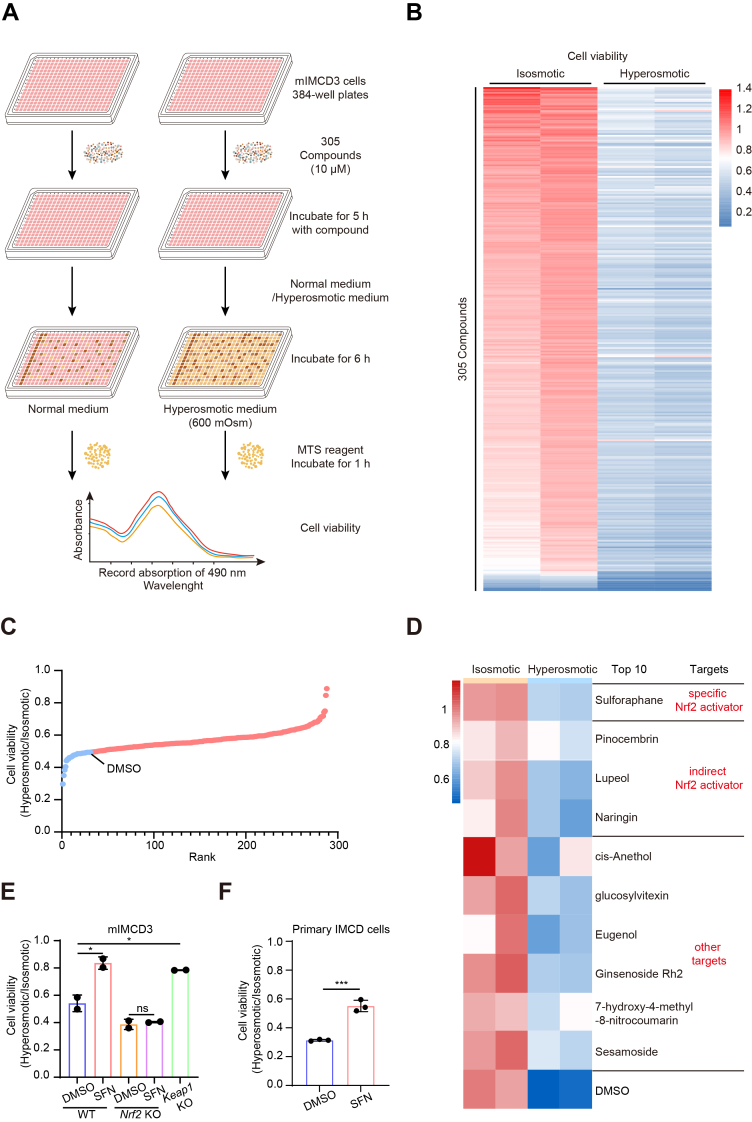
**Compound screening identifies Nrf2 as a regulator of cell viability under hyperosmotic stress.***A*, schematic of the unbiased small-molecule screening strategy in mIMCD3 cells. Cells were pretreated with compounds from the Food as Medicine Compound Library, followed by exposure to isosmotic (300 mOsm) or hyperosmotic (600 mOsm) conditions prior to assessment of cell viability. *B*, heatmap showing cell viability of compound-treated mIMCD3 cells under isosmotic and hyperosmotic conditions. *C*, relative cell viability under hyperosmotic stress, calculated as the ratio of viability under hyperosmotic *versus* isosmotic conditions for each compound. *D*, heatmap of the top 10 compounds conferring the strongest enhancement of cell viability under hyperosmotic stress, ranked based on relative viability in (*C*). Compounds are annotated according to reported molecular targets. *E*, cell viability of wild-type, *Nrf2*-deficient, and *Keap1*-deficient mIMCD3 cells under hyperosmotic stress. *F*, effect of sulforaphane (SFN; 10 μM) on cell viability in primary mouse inner medullary collecting duct (IMCD) cells exposed to hyperosmotic stress.

To identify upstream regulators that enhance cellular survival under hyperosmotic stress, we performed an unbiased small-molecule screen in mIMCD3 cells, a well-established inner medullary collecting duct cell line widely used to study osmotic stress responses (13, 14). Initially, we treated mIMCD3 cells with a library of 305 compounds sourced from the Food as Medicine Compound Library. This library encompasses a diverse collection of food-associated small molecules. After a 5-h incubation with the compounds, the cell culture medium was replaced with either isosmotic (300 mOsm) or hyperosmotic (600 mOsm) medium for 6 hours, after which cell viability was assessed (Fig. 1*A*). Nineteen compounds significantly altered cell viability under isosmotic conditions, including 18 compounds that reduced viability below 0.8 and one compound that increased viability above 1.2 (Figs. 1*B* and S1*A*). These compounds were excluded from subsequent analyses to minimize confounding effects arising from intrinsic cytotoxicity or growth-promoting activity.

As expected, hyperosmotic stress substantially reduced cell viability relative to isosmotic conditions (Fig. 1*B*). To identify small-molecule modulators of cellular survival under hyperosmotic conditions, we calculated and ranked the relative cell viability (hyperosmotic vs. isosmotic) of cells treated with various compounds. As shown in Figure 1*C*, the relative viability of DMSO-treated cells is approximately 0.5. Of the 255 compounds tested, the majority increased cell viability, while 31 reduced it. The top 10 compounds that enhanced viability under hyperosmotic stress were classified according to their known molecular targets (Fig. 1*D*). Notably, four of the top-ranked compounds have been independently reported to activate Nrf2, revealing a non-random enrichment of Nrf2-associated activity among the most effective hits (15, 16, 17, 18, 19). Sulforaphane (SFN), a well-characterized pharmacological activator of Nrf2 (18), was selected as a representative compound for subsequent mechanistic analyses.

To verify whether the protective effect of SFN depends on Nrf2, we generated *Nrf2*-knockout mIMCD3 cells using CRISPR/Cas9 and confirmed the knockout efficiency by Western blot (Fig. S1*B*). Under hyperosmotic stress, SFN failed to enhance viability in *Nrf2*-deficient cells, indicating that the SFN-induced viability enhancement is Nrf2-dependent (Fig. 1*E*). To further rule out potential off-target effects, we adopted an independent genetic approach by knocking out *Keap1*, the key negative regulator of Nrf2 (20). *Keap1* deficiency led to sustained activation of Nrf2, and *Keap1*-deficient cells exhibited a hyperosmotic stress-resistant phenotype (Figs. 1*E* and S1*B*). Together, these complementary genetic approaches demonstrate that Nrf2 activity is both necessary and sufficient to enhance cellular viability under hyperosmotic conditions.

To validate the generality of this regulatory mechanism in primary cells, we isolated primary mouse inner medullary collecting duct (IMCD) cells. Under hyperosmotic stress, SFN treatment significantly enhanced the survival of these primary cells as well (Fig. 1*F*). This result obtained in primary IMCD cells further supports the generality of Nrf2-dependent regulation of cellular responses to hyperosmotic stress.

### Nrf2 promotes osmoprotective transcriptional adaptation to hyperosmotic stress

**Figure 2:**
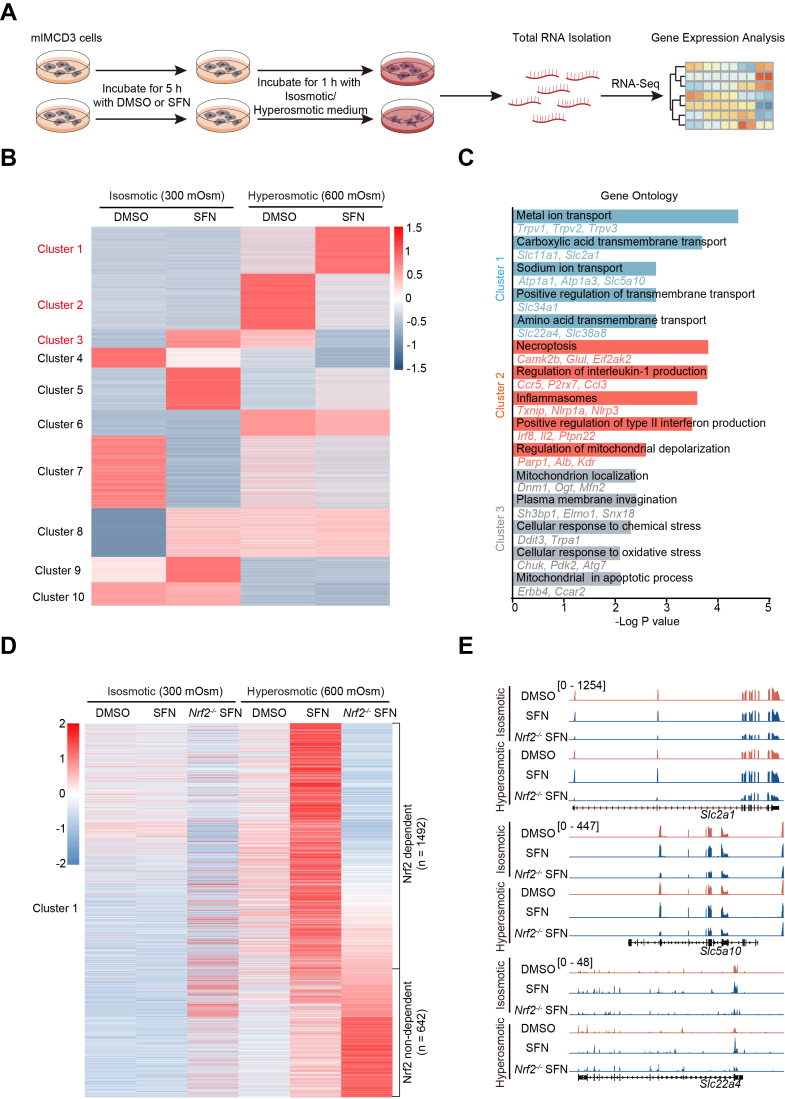
**Nrf2 activation enhances expression of osmoprotective genes in response to hyperosmotic stress.***A*, schematic of the RNA-sequencing experiment in mIMCD3 cells under isosmotic or hyperosmotic conditions. *B*, heatmap of differentially expressed genes (DEGs) clustered by expression patterns across treatment groups. *C*, gene Ontology analysis of genes within Cluster 1, 2, and 3. *D*, heatmap showing RNA expression of Cluster one genes in mIMCD3 cells with indicated treatments. *E*, integrative Genomics Viewer (IGV) snapshot of RNA-seq reads mapping to genomic loci of three representative genes.

To define the transcriptional programs underlying Nrf2-mediated osmotic stress tolerance, we performed RNA sequencing to compare gene expression profiles in cells treated with DMSO or SFN under hyperosmotic conditions (Fig. 2*A*). We found that SFN treatment markedly reshaped the transcriptional landscape under osmotic challenge. Differentially expressed genes were grouped into ten distinct clusters based on their expression patterns (Fig. 2*B*). Under hyperosmotic stress, SFN pretreatment strongly modulated gene expression in clusters 1 to 3, while leaving clusters 4 to 10 largely unaffected. These findings imply that SFN exerts its protective effect primarily through the transcriptional regulation of genes within clusters 1 to 3.

Gene Ontology (GO) analysis showed that genes in cluster one were enriched in pathways critical for osmoprotection, including ion transport, carboxylic acid transmembrane transport, and amino acid transport (Fig. 2*C*). This cluster encompasses genes encoding various transporter proteins, such as Trpv family metal ion channels (Trpv1, Trpv2, Trpv3), the amino acid transporters Slc22a4 and Slc38a8, and sodium ion transporters including Atp1a1, Atp1a3 (subunits of Na^+^/K^+^-ATPase), and Slc5a10. Among these, Trpv1 is known to mediate calcium influx under hyperosmotic stress, elevating intracellular Ca^2+^ levels, which in turn can modulate the activity of other transporters like the Na^+^/K^+^-ATPase to help restore cell volume and electrolyte balance (21). The Na^+^/K^+^-ATPase subunits Atp1a1 and Atp1a3 are essential for establishing the transmembrane electrochemical gradients of Na^+^ and K^+^, which drive osmotic regulation and sodium-coupled solute transport (22, 23, 24). Collectively, these transporters facilitate cellular adaptation to hyperosmotic stress by coordinating ion balance and volume restoration. The upregulation of these genes by SFN treatment likely underlies its survival-promoting effect, leading us to designate cluster 1 as SFN-activated osmoprotective genes.

In contrast, genes in clusters two and three were suppressed by SFN treatment under hyperosmotic stress. GO analysis indicated these clusters were enriched in pathways linked to inflammation and cell death, such as necroptosis, interleukin-1 production, type II interferon production, and regulation of mitochondrial depolarization (Fig. 2*C*). This suggests that SFN enhances cell survival not only by inducing protective transporters but also by concurrently repressing stress-induced death and inflammatory pathways.

To further characterize additional transcriptional programs regulated by SFN, we examined clusters that were prominently induced under isosmotic conditions. Cluster five was strongly activated by SFN treatment and was enriched for oxidative stress, redox homeostasis, and cellular stress response pathways, consistent with a canonical Nrf2-dependent antioxidant transcriptional program (Fig. S2). Cluster 8, which was similarly upregulated by both SFN treatment and hyperosmotic stress, was enriched for pathways related to microtubule organization, cell cycle regulation, and DNA repair (Fig. S2). Given that cluster one showed the most prominent SFN-dependent induction specifically under hyperosmotic conditions and was enriched for osmoprotective functions, we focused our subsequent analyses on this cluster.

We next asked whether SFN-dependent gene activation requires Nrf2. RNA-seq analysis in *Nrf2*-knockout cells revealed that SFN failed to activate approximately 70% of the genes in cluster 1 (1492 of 2134 genes) under hyperosmotic stress (Fig. 2*D*), indicating that the induction of most osmoprotective genes by SFN is Nrf2-dependent. We accordingly defined this subset as Nrf2-dependent osmoprotective genes. Representative RNA-seq tracks for *Slc2a1*, *Slc5a10*, and *Slc22a4* are shown in Figure 2*E*. Taken together, these findings demonstrate that Nrf2 activation protects cells from hyperosmotic stress by orchestrating a transcriptional program that enhances amino acid and ion transport, thereby promoting cellular homeostasis and enhancing cell survival under hyperosmotic stress.

### Nrf2 establishes a permissive chromatin state at osmoprotective loci prior to hyperosmotic challenge

**Figure 3:**
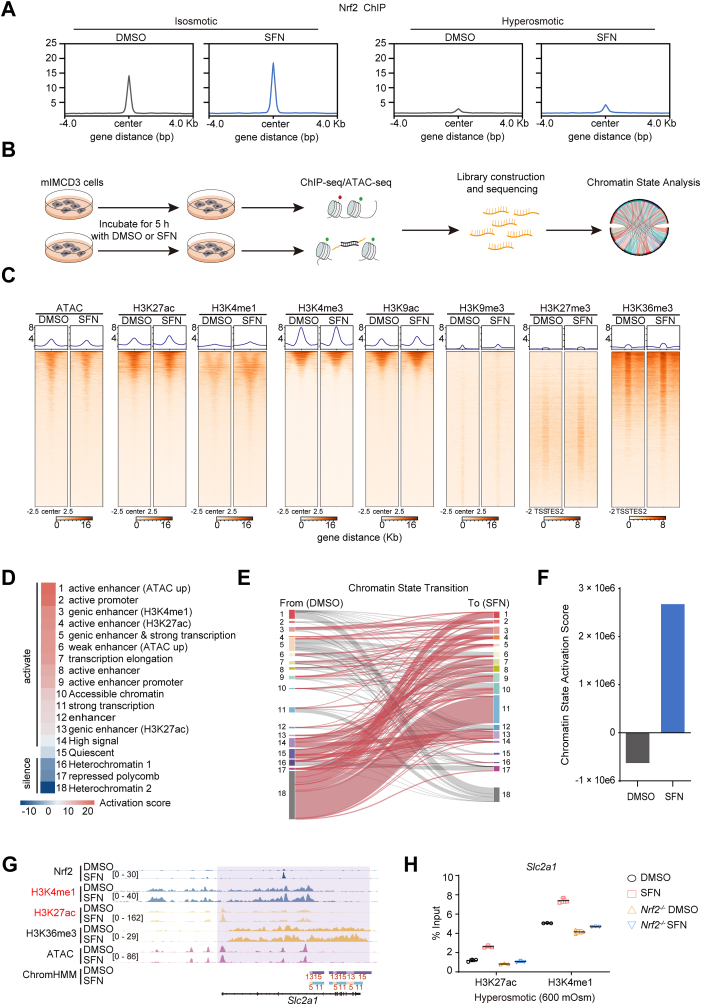
**Nrf2 activation primes the chromatin state of Nrf2-dependent osmoprotective genes.***A*, aggregate Nrf2 ChIP-seq signal centered on Nrf2-binding peaks assigned to Nrf2-dependent osmoprotective genes in mIMCD3 cells under the indicated conditions (±4 kb from peak center). *B*, schematic diagram of ATAC-seq and ChIP-seq analysis in mIMCD3 cells treated with DMSO or SFN. *C*, heatmaps displaying normalized ATAC-seq and ChIP-seq signals for the indicated histone modifications across all detected chromatin regions (peaks centered ± 2.5 kb). *D*, unsupervised clustering of chromatin states derived from combinatorial histone modification patterns, ranked by a composite activation score. *E*, model depicting the SFN-induced chromatin state transition toward a more permissive configuration at Nrf2-dependent osmoprotective genes. *F*, quantification of the chromatin state activation score in mIMCD3 cells treated with DMSO or SFN. *G*, IGV snapshot of ATAC-seq and ChIP-seq tracks for H3K4me1, H3K27ac, and H3K36me3 at the *Slc2a1* locus. *H*, ChIP-qPCR validation of H3K27ac and H3K4me1 enrichment at specific *Slc2a1* regulatory regions in WT or *Nrf2* KO mIMCD3 cells, treated with DMSO or SFN and subjected to 1-h hyperosmotic stress.

To elucidate the mechanism by which Nrf2 activates osmoprotective genes under hyperosmotic stress, we first performed Nrf2 ChIP-seq analysis. The results showed that under basal conditions, SFN treatment increased the binding of Nrf2 to these genes. Unexpectedly, this enhanced Nrf2 binding was not maintained under hyperosmotic stress, when Nrf2 signals at these loci were largely diminished (Fig. 3*A*). Given that SFN enhanced the expression of these genes under hyperosmotic stress, we hypothesized that Nrf2 activation might pre-condition the chromatin state of these genes, thereby priming them for more efficient transcriptional activation under stress.

To test this "chromatin priming" hypothesis, we performed integrative chromatin profiling. We conducted ATAC-seq and ChIP-seq for key histone modifications (H3K27ac, H3K9ac, H3K4me3, H3K4me1, H3K36me3, H3K9me3, and H3K27me3) in cells treated with DMSO or SFN (Fig. 3, *B* and *C*). A random forest model (25) was employed to quantify chromatin activity by integrating these epigenetic marks with gene expression data, where features like ATAC-seq and specific histone modifications (H3K27ac, H3K9ac, H3K4me3, H3K4me1, and H3K36me3) served as active signals, and H3K9me3/H3K27me3 as repressive signals (Fig. S3*A*). Genome-wide chromatin states were systematically annotated using ChromHMM algorithm (26), defining 18 distinct states (Fig. S3*B*).

We focused on chromatin state changes in Nrf2-dependent osmoprotective genes. By evaluating the enrichment patterns of modifications and their regulatory effects on expression, we calculated activation scores for each state and ranked them accordingly. States 1 to 14 were associated with transcriptional activity, whereas states 15 to 18 were linked to silencing, with active enhancers exhibiting the highest scores and heterochromatin the lowest (Fig. 3*D*). Analysis revealed that after SFN treatment, the majority of Nrf2-dependent osmoprotective genes transitioned from more silent states (states 14–18) to highly active states (states 1–11) (Fig. 3*E*).

Quantification using chromatin state activation scores further confirmed that these genes displayed substantially higher scores following SFN treatment, indicating that SFN promotes a shift toward more active chromatin states (Fig. 3*F*). Further analysis revealed that SFN-induced increases in chromatin state activation scores were most pronounced at promoter regions of Nrf2-dependent osmoprotective loci under isosmotic conditions (Fig. S4). Taking the osmoprotective gene *Slc2a1* as an example, SFN treatment markedly increased the levels of H3K27ac and H3K4me1 modifications at its promoter and enhancer regions, accompanied by a transition toward more active chromatin states, visually illustrating the chromatin state remodeling induced by SFN (Fig. 3*G*). Importantly, this SFN-induced active chromatin state was maintained even under hyperosmotic stress but was completely absent in *Nrf2*-knockout cells (Fig. 3*H*). Collectively, these findings indicate that Nrf2 licenses transcriptional competence through chromatin remodeling rather than acting as a sustained DNA-bound activator during osmotic stress.

### Nrf2-dependent chromatin priming enables efficient NFAT5 recruitment during osmotic stress

Given that active chromatin is more accessible for transcription factor recruitment (27, 28), we hypothesized that Nrf2-mediated chromatin activation facilitates the binding of osmosensitive transcription factors to the loci of osmoprotective genes. Nfat5 (also known as TonEBP) is the most critical transcription factor responding to hyperosmotic stress, responsible for activating numerous osmoprotective genes to maintain cellular homeostasis (2, 29, 30, 31). Assessment of Nfat5 expression revealed that SFN treatment did not cause notable changes in either mRNA or protein levels under normal conditions or hyperosmotic stress (Fig. S5, *A* and *B*).

**Figure 4:**
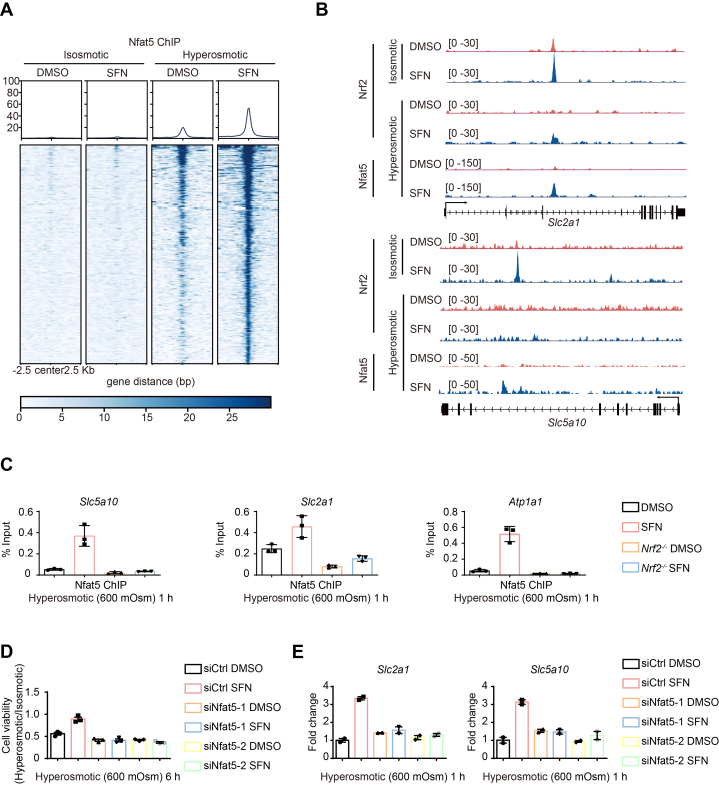
**Nrf2 facilitates the binding of Nfat5 to Nrf2-dependent osmoprotective genes.***A*, aggregate Nfat5 ChlP-seq signal centered on Nfat5-binding peaks assigned to Nrf2-dependent osmoprotective genes in mIMCD3 cells under the indicated conditions (peaks centered ± 2.5 kb). *B*, IGV snapshot of Nrf2 and Nfat5 ChIP-seq tracks at the loci of two representative genes, *Slc2a1* and *Slc5a10*. *C*, ChIP-qPCR quantification of Nfat5 enrichment at specified genomic loci in WT or *Nrf2* KO mIMCD3 cells, pretreated with DMSO or SFN before a 1-h hyperosmotic challenge. *D*, cell viability of mIMCD3 cells under hyperosmotic stress, treated with 10 μM SFN or DMSO, in the presence or absence of Nfat5 knockdown. *E*, RT-qPCR analysis of *Slc5a10* and *Slc2a1* mRNA expression in mIMCD3 cells under hyperosmotic stress, comparing control and Nfat5-knockdown conditions.

Notably, Nfat5 ChIP-seq analysis demonstrated that SFN treatment greatly enhanced the binding of Nfat5 to Nrf2-dependent osmoprotective genes under hyperosmotic stress (Fig. 4*A*). Taking the *Slc2a1* and *Slc5a10* genes as examples, their ChIP-seq signal tracks visually demonstrated that SFN treatment substantially increased the Nfat5 binding peaks (Fig. 4*B*). Quantitative results from ChIP-qPCR at three gene loci - *Slc2a1*, *Slc5a10*, and *Atp1a1*—further validated this enhancement effect (Fig. 4*C*). Most importantly, in *Nrf2*-knockout cells, the SFN-induced enhancement of Nfat5 binding was completely abolished (Fig. 4*C*). These data indicate that the active chromatin environment established by Nrf2 activation is a prerequisite for the efficient recruitment of Nfat5 to target genes under hyperosmotic stress.

We next explored whether the protective effects of SFN against hyperosmotic stress are mediated by enhanced recruitment of Nfat5 to osmoprotective genes. We first knocked down Nfat5 in mIMCD3 cells using two effective Nfat5 siRNAs (Fig. S5, *C* and *D*) and then assessed the impact of this knockdown on the protective effects of SFN against hyperosmotic stress. As shown in Figure 4*D*, SFN significantly increased cell viability under hyperosmotic stress in control cells, but this effect was lost in *Nfat5*-knockdown cells. Additionally, *Nfat5* knockdown prevented the SFN-induced expression of Nrf2-dependent osmoprotective genes (Fig. 4*E*). These data indicate that Nfat5 is a critical mediator of SFN-conferred protection against hyperosmotic stress.

Collectively, these findings suggest that SFN-induced Nrf2 activation modifies the chromatin state of osmoprotective genes, enabling enhanced binding of Nfat5 under hyperosmotic stress and thereby boosting the expression of osmoprotective genes.

### Nrf2 promotes renal osmotic tolerance and tissue protection during dehydration *in vivo*

**Figure 5:**
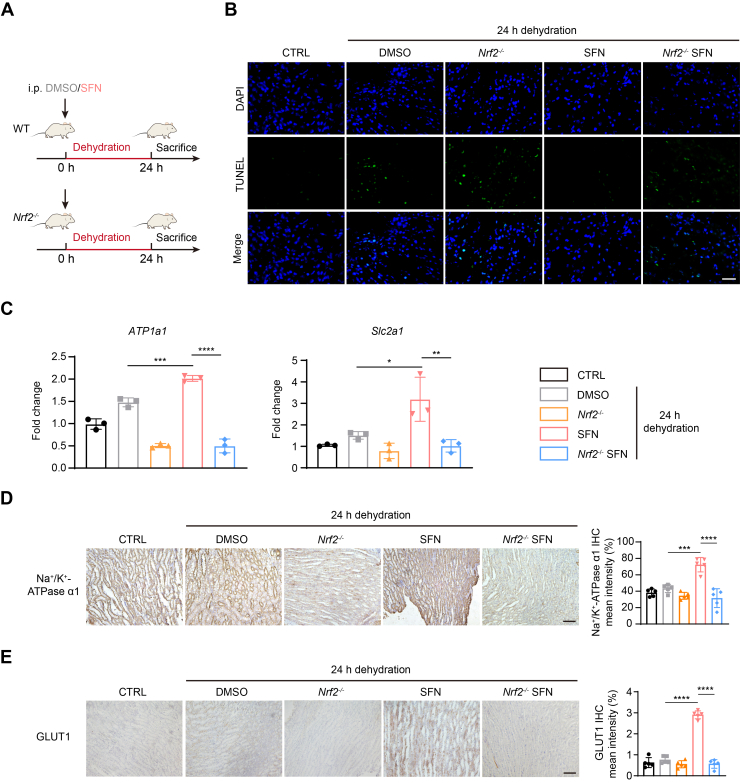
**Pharmacological activation of Nrf2 attenuates dehydration-induced renal injury *via* upregulation of osmoprotective genes.***A*, experimental design for the *in vivo* dehydration challenge. Wild-type (WT) and *Nrf2*^−/−^ mice, with or without SFN pretreatment, were subjected to 24-h water deprivation. *B*, assessment of renal cell death by TUNEL staining. *C*, RT-qPCR analysis of mRNA levels for the osmoprotective genes *Atp1a1* and *Slc2a1* in mouse renal inner medulla tissue with indicated treatments. *D*, immunohistochemical staining and quantification of Na^+^/K^+^-ATPase α1 (encoded by *Atp1a1*) in renal tissue. *Left*: representative images. *Right*: quantification of signal intensity. *E*, immunohistochemical staining and quantification of glucose transporter 1 (GLUT1, encoded by *Slc2a1*) in renal tissue. *Left*: representative images. *Right*: quantification of signal intensity. *C–E*, data are presented as mean ± SD. One-way ANOVA was used for all statistical analysis. ∗*p* < 0.05, ∗∗*p* < 0.01, ∗∗∗*p* < 0.001, ∗∗∗∗*p* < 0.0001.

To determine whether Nrf2-mediated regulation of osmotic stress–responsive transcription confers physiological protection *in vivo*, we subjected wild-type and Nrf2-deficient mice to dehydration-induced osmotic challenge. C57BL/6 wild-type (WT) and *Nrf2*^*−/−*^ mice were subjected to 24-h water deprivation (Fig. 5*A*), a condition that induces systemic dehydration and markedly elevates the osmolarity of the renal medullary interstitium, thereby exposing renal epithelial cells to profound hyperosmotic stress. Under these conditions, *Nrf2*^*−/−*^ mice exhibited a pronounced increase in renal cell death compared with WT controls, as assessed by TUNEL staining (Fig. 5*B*), indicating an essential role for Nrf2 in maintaining cellular survival within the hyperosmotic renal microenvironment.

To evaluate the renoprotective potential of pharmacologically activated Nrf2, mice were pretreated with SFN before water deprivation. SFN treatment significantly attenuated dehydration-induced renal cell death in WT mice, an effect that was entirely absent in *Nrf2*^*−/−*^ mice (Fig. 5*B*). Mechanistically, RT-qPCR analysis of mouse renal inner medulla tissue showed that SFN induced the expression of key osmoprotective genes, including *Atp1a1* and *Slc2a1*, in an Nrf2-dependent manner (Fig. 5*C*). This transcriptional upregulation was confirmed at the protein level by immunohistochemistry, which revealed increased abundance of Na^+^/K^+^-ATPase α1 (encoded by *Atp1a1*) and glucose transporter 1 (GLUT1, encoded by *Slc2a1*) specifically in the kidneys of SFN-treated WT, but not *Nrf2*^*−/−*^, mice (Fig. 5, *D* and *E*). Collectively, these results demonstrate that Nrf2 activation bolsters renal osmotic tolerance and preserves tissue integrity during dehydration by orchestrating the expression of osmoprotective genes.

**Figure 6:**
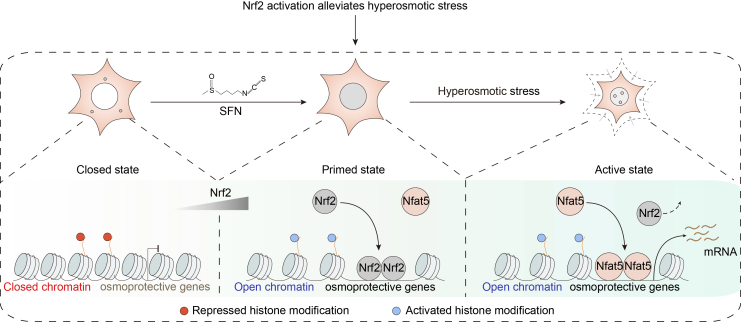
**Working model for Nrf2-mediated chromatin priming in osmotic stress adaptation.** Under basal conditions, osmoprotective gene loci are characterized by relatively inactive chromatin states and *low* transcriptional output. Activation of Nrf2 establishes activation-associated chromatin states and activating histone modifications at NFAT5 target loci without directly inducing transcription. Upon hyperosmotic stress, NFAT5 is more efficiently recruited to these primed loci, resulting in amplified NFAT5-dependent transcription and enhanced osmotic stress tolerance.

Collectively, our findings support a model in which Nrf2 enhances osmotic stress -responsive transcription through a mechanism distinct from direct transcriptional activation. Rather than sustaining transcription during hyperosmotic stress or altering NFAT5 abundance or activation, Nrf2 promotes a transition toward more active chromatin states at NFAT5 target loci prior to stress exposure. This chromatin-based priming facilitates more efficient NFAT5 recruitment upon hyperosmotic challenge, thereby amplifying NFAT5-dependent transcription and supporting cellular adaptation to osmotic stress (Fig. 6).

## Discussion

Hyperosmotic stress poses a fundamental challenge to cellular homeostasis, and transcriptional adaptation has traditionally been viewed as a stimulus-driven process dominated by stress-responsive transcription factors such as NFAT5 (2, 8). Here, we identify Nrf2 as a previously unrecognized upstream regulator of osmotic stress - responsive transcription that operates through a distinct chromatin-based mechanism. Rather than acting as a sustained transcriptional activator during osmotic stress, Nrf2 functions as an epigenetic priming factor that establishes a permissive chromatin landscape at osmoprotective gene loci prior to hyperosmotic challenge, thereby licensing efficient transcriptional activation upon stress exposure. Notably, this priming effect persists even when Nrf2 occupancy at these loci is diminished during stress, uncoupling chromatin licensing from stimulus-dependent transcriptional activation.

Our findings define a hierarchical regulatory framework in which Nrf2 acts upstream of NFAT5 to shape transcriptional competence. While NFAT5 remains the principal driver of osmoprotective gene transcription in response to hyperosmotic stress, the efficiency of NFAT5 recruitment and transcriptional output is critically determined by the underlying chromatin state (32, 33). By establishing activation-associated chromatin states, including increased H3K9ac, H3K27ac and H3K4me1 enrichment at NFAT5 target loci, Nrf2-dependent priming enhances NFAT5 binding without altering NFAT5 expression or abundance. This separation of chromatin state regulation from transcription factor activation provides a mechanistic explanation for how cells fine-tune the magnitude and robustness of transcriptional responses under osmotic challenge.

More broadly, this study highlights chromatin state regulation as a mechanism that enhances cellular preparedness for environmental stress. By decoupling chromatin state establishment from stimulus-induced transcription factor activation, cells can preconfigure stress-responsive loci to enable rapid and amplified transcriptional responses when challenges arise. Such chromatin-based licensing mechanisms may extend beyond osmotic stress, providing a general framework through which stress-responsive transcription factors integrate chromatin context with environmental cues to modulate transcriptional output. In this regard, Nrf2 exemplifies a class of regulators that shape future transcriptional potential rather than directly executing stress-induced gene expression programs.

The physiological relevance of this regulatory mechanism is supported by our *in vivo* findings. In a dehydration-induced hyperosmotic model, renal epithelial cells exhibited increased cell death, which was further exacerbated in Nrf2-deficient mice. Conversely, pharmacological activation of Nrf2 restored osmoprotective gene expression and significantly reduced renal injury. These results establish a functional link between chromatin-based transcriptional priming and tissue-level resilience to hyperosmotic stress *in vivo*.

Nrf2 is best known as a master regulator of antioxidant and cytoprotective responses and has emerged as an important suppressor of ferroptosis through the regulation of genes involved in redox homeostasis, lipid peroxide detoxification, and iron metabolism. Recent studies have further expanded this framework by demonstrating roles for Nrf2 in ferritinophagy and iron homeostasis, highlighting its broader contribution to iron–lipid metabolic regulation (34, 35, 36, 37). Within this context, our findings identify an additional layer of Nrf2 function. Rather than directly activating osmoprotective genes during hyperosmotic stress, Nrf2 promotes activation-associated chromatin states before stress exposure, thereby facilitating efficient NFAT5-dependent transcriptional responses during subsequent osmotic challenge.

Importantly, our work expands the functional repertoire of Nrf2 beyond its canonical antioxidant and cytoprotective roles. Rather than acting solely as a stress-induced transcriptional activator, Nrf2 emerges here as a chromatin-associated regulator capable of shaping transcriptional competence in advance of environmental challenge. This mode of action – modulation of chromatin states rather than direct gene induction - suggests a broader principle by which cells integrate metabolic and stress-related signals to fine-tune adaptive gene regulatory programs.

Several limitations of this study should be acknowledged. First, although our data demonstrate that Nrf2 promotes activation-associated chromatin states at osmoprotective loci, the chromatin-modifying cofactors responsible for these epigenetic changes remain unknown. Second, while we observed reduced Nrf2 occupancy following hyperosmotic stress, the molecular mechanisms underlying this redistribution and its functional significance require further investigation. Finally, it will be important to establish whether similar Nrf2-dependent chromatin regulatory mechanisms operate in other stress-responsive pathways beyond osmotic adaptation.

In summary, this study uncovers a previously unrecognized epigenetic role for Nrf2 in osmotic stress adaptation. By promoting activation-associated chromatin states at osmoprotective gene loci, Nrf2 facilitates NFAT5-dependent transcription and promotes cellular survival under hyperosmotic conditions. Together, these findings provide new insight into the chromatin-based regulation of osmotic homeostasis and establish a conceptual framework for understanding how transcriptional competence is preconfigured in anticipation of environmental stress.

## Materials and methods

### Study design

Initially identified through an unbiased high-throughput chemical screen, this study aimed to elucidate the mechanism by which Nrf2 activation enhances cellular survival under hyperosmotic stress. The drug screening experiments data were from two biological replicates. The mechanism of Nrf2-dependent chromatin and gene expression changes was assessed through ChIP-seq, ATAC-seq, and RNA-seq. ChIP-seq and ATAC-seq data were derived from a single biological replicate, and RNA-seq data were obtained from two biological replicates. The RNA-seq, ChIP-seq and ATAC-seq data from this study are available in the Gene Expression Omnibus (GEO) database and can be accessed using the access token “krkrkkmqtbqlpwb” (GSE266024).

### Mice and drug treatment

*Nrf2* KO mice were provided by Jingbo Pi (School of Public Health, China Medical University). The mice were housed in a controlled environment with a 12-h light-dark cycle, temperatures between 21 to 25 °C, and humidity levels of 30 to 70%. They had ad libitum access to food and water. Eight-week-old male mice were used for the experiments and randomly assigned to experimental groups. All animals had free access to food and were either allowed free access to water or subjected to 24 h of water restriction. For sulforaphane treatment, mice received intraperitoneal injections of sulforaphane (10 mg/kg/day, dissolved in saline; LKT Labs, S8047) prior to water restriction, while control mice were injected with DMSO. After treatment, mice were perfused with PBS and euthanized. The left kidneys were embedded in OCT for cryosectioning, and the renal medulla from the right kidneys was collected for further analysis. All mouse care and experimental protocols were approved by the Ethical Committee of Tianjin Medical University (TMUaMEC 2022016).

### Cell culture

mIMCD3 cells and primary IMCD cells were cultured in Dulbecco's Modified Eagle Medium/Nutrient Mixture F-12 (DMEM/F12) medium containing 10% fetal bovine serum (FBS) at 37 °C in a humidified 5% CO2 atmosphere.

### Primary IMCD cell isolation from mouse kidneys

Primary renal epithelial cells were isolated from the kidneys of healthy mice, and DBA^+^ cells were enriched following a previously established protocol (38). Briefly, kidney tissues underwent enzymatic digestion with collagenase/hyaluronidase (07,919, STEMCELL Technologies) at 37 °C for 90 min. The cell suspension was then incubated with biotinylated anti-DBA antibody (B-1035, Vector Laboratories) at 4 °C for 10 min, followed by purification using the Collection Biotin Binder Kit (11533D, Invitrogen) at room temperature. The purified DBA^+^ cells were subsequently immortalized by transduction with SV40 large T antigen (Genomeditech) to establish a DBA^+^ immortalized cell line.

### Drug screening

The drug screening experiment was conducted at Tianjin Medical University's Basic Medical Research Center using the Explorer G3 automated system and a high-throughput screening platform library. mIMCD3 cells were plated in 384-well plates (1500 cells/well) on day 0 and cultured overnight. On day 1, compounds (10 μM) were applied in duplicate. After 5 hours of treatment, cells were cultured in either isosmotic or hyperosmotic medium (DMEM/F12 with 120 mM NaCl and 80 mM urea) for 6 hours. Cell viability was assessed by adding MTS reagent (Promega, G3581) and measuring absorbance at 490 nm.

### Generation of *Nrf2* knock out and *Keap1* knock out cell line

LentiGuide-Puro (#52963) and LentiCas9-Blast (#52962) constructs were obtained from Addgene, sgRNAs were designed using sgRNA Designer (https://portals.broadinstitute.org/gpp/public/analysis-tools/sgrna-design). To generate the *Nrf2* knock out and *Keap1* knock out cell line, cells were infected with appropriate amounts of lentiviral particles for 12 h, and then virus-containing medium was removed and replaced with fresh medium for 48 h. Stably transduced cells were selected by blasticidin. Stable cells expressing Cas9 was infected and selected with puromycin. The expression of Nrf2 and Keap1 were detected *via* immunoblotting.

### ATAC-seq

100,000 cells were lysed in a lysis buffer (containing 10 mM Tris–HCl, pH 7.4, 10 mM NaCl, 3 mM MgCl2, and 0.1% (v/v) IGPAL CA-630) for 10 min on ice, followed by centrifugation at 500*g* for 5 min. The isolated nuclei were supplemented with 50 μl of transposition reaction buffer (comprising 5 μl of TruePrep Tagment Enzyme, 10 μl of TruePrep Tagment Buffer L, and 35 μl of ddH2O from Vazyme TD501–01) and then incubated at 37 °C for 30 min. After tagmentation, VAHTS DNAClean Beads were employed to halt the reaction, and DNA was subsequently purified for the final library construction using the TruePrepTM DNA Library Prep Kit V2 for Illumina. Finally, paired-end high-throughput sequencing was carried out using the HiSeq X Ten platform.

### Chromatin immunoprecipitation sequencing and ChIP-qPCR

mIMCD3 cells were fixed with 1% formaldehyde at room temperature for 10 min. Crosslinking was halted by adding 150 mM of glycine for 5 min at room temperature. Subsequently, cells were washed twice with PBS and harvested using chromatin immunoprecipitation (ChIP) cell lysis buffer (containing 10 mM EDTA, 1% SDS, and 50 mM Tris-HCl at pH 8.0). The cells were then fragmented by sonication (ranging from 200 to 500 bp). Immunoprecipitation was carried out using 2 μg of NRF2 (Abcam, ab62352), NFAT5 (Abcam, ab3446), H3K27ac (Abcam, ab4729), H3K4me1 (Abcam, ab8895), H3K4me3 (Millipore, 05–745R), H3K9ac (Abcam, ab4441), H3K9me3 (Abcam, ab8898), H3K27me3 (Abcam, ab6002) and H3K36me3 (Abcam, ab9050) antibodies. After elution and reversal of crosslinking, samples were treated with RNase A for 30 min at 37 °C. For ChIP-qPCR, 1 μl purified DNA was used per qPCR reaction (Table S1).

### RNA sequencing and real-time quantitative PCR

Total RNA was extracted from cells or kidney tissues using TRIzol. RNA sequencing (RNA-seq) was conducted on the Illumina NovaSeq platform. Hisat2 was utilized to align sequence reads to the mm10 reference genome sequence. Samtools was employed to sort the aligned reads, and read counts per gene were acquired from the mapped reads through FeatureCounts. Mfuzz algorithm were used to analyze dynamic gene expression. All sequencing data have been deposited in the Gene Expression Omnibus (GSE266023). For real-time quantitative PCR (RT-qPCR), the total RNA was reverse transcribed into complementary DNA (cDNA) utilizing the cDNA Synthesis Kit. Real-time quantitative polymerase chain reaction (RT-qPCR) was conducted employing SYBR Green Master along with gene-specific primers (Table S2).

### ChIP-seq and ATAC-seq analysis

The Bowtie2 algorithm was employed to align paired-end reads obtained from ChIP-seq and ATAC-seq experiments to the mouse reference genome mm10. To eliminate PCR duplicates and reads aligned to mtDNA, the "mark duplicates" command from Picard (http://broadinstitute.github.io/picard/) was utilized. Subsequently, ChIP-seq and ATAC-seq peaks were identified using HOMER's findPeaks algorithm. Bigwig files were generated based on the deepTools output. Statistical differences in normalized read counts across peaks between sets of ChIP-seq experiments were determined using HOMER's getDifferentialPeaks.pl, which leverages the statistical modeling functions of DESeq2. ChromHMM algorithm was then applied to define chromatin states. ChIP-seq peaks were first called using HOMER and converted into binarized presence/absence matrices using the ChromHMM BinarizeBed function with 200-bp genomic bins. ChromHMM was then used to learn combinatorial chromatin states based on the distribution of histone modification signals across the genome. For downstream analyses, chromatin activity was quantified using state-specific activation scores derived from the enrichment patterns of activation-associated histone marks. Activity scores for promoters, enhancers, and gene bodies were calculated as the length-weighted sum of chromatin states overlapping each genomic region. The AnnotatePeaks.pl algorithm was utilized to analyze the distribution of peaks around the transcription start site (TSS). Genomic data have been deposited in the Gene Expression Omnibus (GSE266021 and GSE266022).

### TUNEL

TUNEL staining was performed on frozen kidney sections using a YF488 TUNEL Apoptosis Detection Kit (T6013S, UElandy, China). Briefly, 50 μl of TUNEL reaction mixture containing TdT enzyme and reaction buffer was applied to each section and incubated at 37 °C for 1 h in the dark. The reaction mixture was then removed, and the sections were washed twice with PBS. Finally, the slides were counterstained with DAPI and mounted for imaging.

### Immunohistochemistry

The mouse kidney tissues were perfused with 10% formalin overnight and subsequently embedded in paraffin. Tissue sections were treated with 3% H2O2 for 15 min, followed by blocking with 5% BSA for 1 h at room temperature. Kidney sections were then subjected to incubation with primary antibodies against Atp1a1 (14418-1-AP, Proteintech) and Glut1 (sc-377228, Santa cruz). Following primary antibody incubation, sections were further incubated with anti-mouse/rabbit HRP secondary antibody (ZSGB-BIO pv-6000).

### Immunoblotting

For Western blot analysis, cells were lysed in radioimmunoprecipitation assay lysis buffer containing a phosphatase inhibitor cocktail, a protease inhibitor cocktail, and dithiothreitol. The antibodies used in this study included NRF2 (Abcam, ab62352), α-tubulin (Proteintech, 11224-1-AP) and NFAT5 (Abcam, ab3446).

### Statistical analysis

Significance was assessed using a two-tailed unpaired *t* test or One-way ANOVA to compare two independent groups, analyzed with GraphPad Prism Software. A *p*-value < 0.05 was considered statistically significant. Results are presented as means ± SD, based on data from at least two independent experiments.

### Data availability

The RNA-seq, ChIP-seq and ATAC-seq data from this study are available in the Gene Expression Omnibus (GEO) database and can be accessed using the access token “krkrkkmqtbqlpwb” (GSE266024). All data necessary to evaluate the conclusions in this paper are included within the paper and/or the Supporting Information.

## Supporting information

This article contains supporting information.

## Conflict of interest

The authors declare that they have no conflicts of interest with the contents of this article.
